# Synergistic Effect of Chemical Penetration Enhancers on Lidocaine Permeability Revealed by Coarse-Grained Molecular Dynamics Simulations

**DOI:** 10.3390/membranes11060410

**Published:** 2021-05-29

**Authors:** Marine E. Bozdaganyan, Philipp S. Orekhov

**Affiliations:** 1 School of Biology, Lomonosov Moscow State University, 119234 Moscow, Russia; bozdaganyan@mail.bio.msu.ru; 2 N.N. Semenov Federal Research Center for Chemical Physics, Russian Academy of Sciences, 119334 Moscow, Russia; 3 Institute of Personalized Medicine, Sechenov University, 119991 Moscow, Russia; 4 Research Center of Molecular Mechanisms of Aging and Age-Related Diseases, Moscow Institute of Physics and Technology, 141701 Dolgoprudny, Russia

**Keywords:** chemical penetration enhancers, transdermal drug delivery, stratum corneum, molecular dynamics simulations, coarse-grained simulations

## Abstract

The search for new formulations for transdermal drug delivery (TDD) is an important field in medicine and cosmetology. Molecules with specific physicochemical properties which can increase the permeability of active ingredients across the stratum corneum (SC) are called chemical penetration enhancers (CPEs), and it was shown that some CPEs can act synergistically. In this study, we performed coarse-grained (CG) molecular dynamics (MD) simulations of the lidocaine delivery facilitated by two CPEs—linoleic acid (LA) and ethanol—through the SC model membrane containing cholesterol, N-Stearoylsphingosine (DCPE), and behenic acid. In our simulations, we probed the effects of individual CPEs as well as their combination on various properties of the SC membrane and the lidocaine penetration across it. We demonstrated that the addition of both CPEs decreases the membrane thickness and the order parameters of the DPCE hydrocarbon chains. Moreover, LA also enhances diffusion of the SC membrane components, especially cholesterol. The estimated potential of mean force (PMF) profiles for the lidocaine translocation across SC in the presence/absence of two individual CPEs and their combination demonstrated that while ethanol lowers the free energy barrier for lidocaine to enter SC, LA decreases the depth of the free energy minima for lidocaine inside SC. These two effects supposedly result in synergistic penetration enhancement of drugs. Altogether, the present simulations provide a detailed molecular picture of CPEs’ action and their synergistic effect on the penetration of small molecular weight therapeutics that can be beneficial for the design of novel drug and cosmetics formulations.

## 1. Introduction

Transdermal drug delivery (TDD) is a method of delivering drugs systemically by applying a drug formulation onto intact and healthy skin [1]. Stratum corneum (SC) serves as a rate-limiting lipophilic barrier against the uptake of chemical and biological toxins as well as transepidermal water loss [2]. The structure of SC is organized as stacked bilayers of ceramides in a splayed chain conformation with cholesterol associated with the ceramide sphingoid moiety, and free fatty acids associated with the ceramide fatty acid moiety [3]. Only a minority of molecules with specific physico-chemical properties can cross the skin sufficiently, and in the case of drugs with the blood circulation target subdermal tissue [4]. There are different approaches which are used in TDD to overcome the skin barrier: physical (e.g., iontophoresis, sonophoresis, electroporation, microfabricated microneedles, local temperature increase) [5], chemical (use of penetration enhancers) [6], and the use of carriers (vesicles and micro/nanoparticles) [7]. Regardless of the specific TDD approach, the drug initially penetrates through the SC, and then passes through the deeper epidermis and dermis without drug accumulation in the dermal layer. When a drug reaches the dermal layer, it becomes available for systemic absorption via the dermal microcirculation [3,8]. 

Chemical penetration enhancers (CPE) are proven to increase the transport of drugs across the skin layers. They achieve their effects by different mechanisms that depend on the chemical nature of penetration enhancers and the properties of the SC, which can be changed dramatically [4,6,9,10,11,12,13,14,15]. The molecular mechanisms of CPE action on the membrane were widely studied by molecular dynamics simulations [16,17,18,19,20,21,22,23,24,25,26,27,28,29,30,31,32,33,34,35]. In the recent study, the authors [36] analyzed permeability properties for several CPEs and built a model for prediction of permeability coefficients. It was shown that the polar groups of fatty acids serving as CPEs were associated with the ceramide headgroup region while the apolar tails were generally aligned with the ceramide chains. The different shapes of the penetration enhancer molecules, and their degree of saturation, determine to what extent they disrupt the skin barrier lipid packing. [36]. Additionally, in the work of [37], it was shown that ethanol enhanced bilayer permeability by both mechanisms—extraction of the skin lipids and enhancing the mobility of lipid chains.

Synergistic combinations of chemical penetration enhancers result in a higher permeability for the active ingredient than the average of activities of individual enhancers and depends on the physico-chemical properties, concentration and ratio used in the experiment [3,38,39,40,41]. Synergistic effects of different CPEs have been described in several papers [13,14,15,38,42]. Particularly, a method of in vitro skin impedance guided high-throughput (INSIGHT) screening was created and used to explore synergistic mixtures of CPEs which could deliver active ingredients across the skin [15]. The possible mechanisms of synergistic actions were discussed in [14,43,44]. The most frequently used enhancer combinations include fatty acids with propylene glycol, terpenes with propylene glycol, and fatty acids with ethanol [45]. The latter formulation has been chosen in the present study to demonstrate the synergistic effect on the permeability of lidocaine, one of the most popular local anesthetics used for a variety of medical procedures including treatment of open skin sores, lesions, and in surgical procedures [46,47]. Lidocaine is an amphiphilic molecule which can penetrate the SC membrane simultaneously [48,49] or at higher rates when applied with various CPEs [38,46,50,51,52,53]. 

In the work of [38], it was demonstrated that linoleic acid (LA) and ethanol (EtOH) formulation was the most effective of tested enhancers, increasing the lidocaine flux by 42-fold compared to that from PBS. It was suggested that bilayer disordering agents, such as linoleic acid and ultrasound, transform the SC lipid bilayers into a fluid lipid bilayer phase or create a separate bulk oil phase [38]. 

In the present study, we investigated the molecular mechanism by which LA/EtOH formulation can synergistically enhance the delivery of lidocaine. We have carried out the coarse-grained (CG) molecular dynamics (MD) simulations of fully hydrated SC model membrane with lidocaine (LID) in presence and absence of LA and EtOH. The effect of ethanol and linoleic acid on skin lipid bilayer was explained in both structural properties and free energy changes of lidocaine translocation through the membrane.

## 2. Materials and Methods

### 2.1. Coarse-Grained Models of Ethanol, Linoleic Acid, Lidocaine and SC Membrane

**Figure 1:**
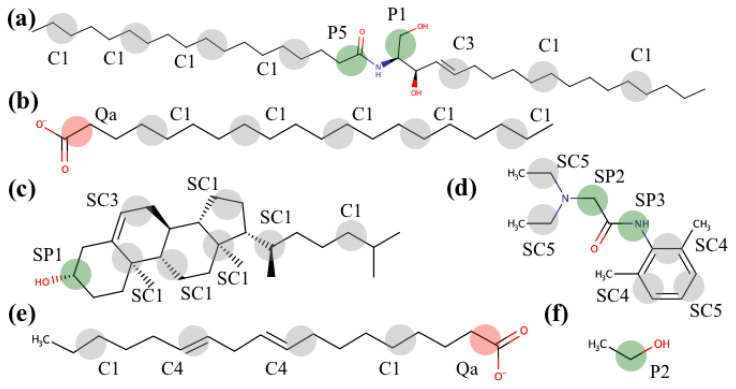
Structural formulae of the SC membrane components: DPCE (**a**), behenic acid (**b**), cholesterol (**c**); lidocaine (**d**); penetration enhancers: linoleic acid (**e**) and ethanol (**f**). Coarse-grained beads and their corresponding MARTINI force field types are also shown schematically.

In the present study, we utilized the popular MARTINI force field for the coarse-grained simulations, which has proved itself as a useful tool for a variety of applications including investigations of interaction between chemical compounds of different nature and biological membranes, membrane pore formation, fusion and disruption, lipid phase transitions, effects of CPEs and detergents on membrane properties, and many more [54,55]. Despite certain limitations of this force field, which does not account explicitly for hydrogen bonds, proper distribution of partial charges, smoother stereochemical interactions resulting in faster diffusion [56], and stickier interactions between proteins (mostly relevant to soluble proteins [57]), it describes the interactions between amphiphilic compounds and biological membranes satisfactorily [58]. The standard MARTINI library already contains molecular topologies for DPCE, cholesterol, behenic acid, and ethanol (see Figure 1). The coarse-grained models for lidocaine and linoleic acid (see Figure 1d–e) were developed based on the auxiliary all-atom simulations according to the iterative scheme we used before [59] until satisfactory agreement was reached between the AA and CG models. In order to select MARTINI CG particle types, we applied the automated toolkit [60] and referred to the already parameterized molecules. The resulting topologies are available at https://github.com/porekhov/cg_topologies.

The reference all-atom simulations were run for 100 ns in the NPT ensemble (T = 320 K maintained by the V-rescale algorithm, P = 1 bar controlled by Parrinello–Rahman barostat; the integration time step = 2 fs; the Verlet cutoff scheme and particle mesh Ewald (PME) were used for the nonbonded interactions with the cutoff value set to 1.2 nm).

### 2.2. Details of Coarse-Grained Simulations and Analysis

The initial models of SC membranes were assembled using CHARMM-GUI web service [61]. Further, insane.py [62] was used to solvate the systems. Before running any MD simulations, the steepest descent minimization was performed. The details about all of the simulated systems (simulation time, composition) are provided in Table 1. 

The simulation parameters were chosen based on recommendations [63]. The systems were simulated in the NPT ensemble using the V-rescale thermostat (T = 320 K, τ_t_ = 1.0 ps) and the Parrinello–Rahman barostat (time constant = 12.0 ps, compressibility = 3 × 10^−4^ bar^−1^, applied semi-isotropically). All CG simulations were performed with the polarizable water model [64] and the reaction field approach for the long-range electrostatics (ε_r_ = 2.5). The time step was 20 fs. Gromacs 2019.4 was utilized for all simulations [65].

To estimate the potential of mean force (PMF) profiles, we employed a scheme similar to one used before [66]. Briefly, the lidocaine molecule was slowly pulled with the constant speed (pulling speed = 1 × 10^−5^ nm⋅ps^−1^; force constant of pulling harmonic potential = 2000 kJ⋅mol^−1^⋅nm^−2^) toward the center of SC membrane and the resulting PMF was obtained by integration of the instant force applied along the simulation time.

The membrane density profiles, thickness, diffusion coefficients of the lipids and order parameters for the ceramide chains were calculated as it is described in [67], using Python scripts exploiting the MDAnalysis toolkit [68], and are available upon request.

## 3. Results

### 3.1. Equilibrium MD Simulations of Lidocaine in the Absence and Presence of the CPEs

Several MD systems were developed including SC model membrane with 50% EtOH, SC model membrane with LA, and membrane with LA and 50% EtOH; as control, we used the same SC model membranes which were solvated in the explicit coarse-grained 0.15M NaCl solution. We have further analyzed the properties of the lipid bilayers and the effects of CPEs on them based on the equilibrium 1-μs long simulations: density of individual SC membrane components, membrane thickness, and diffusion coefficients of lipids and fatty acids. 

As it was previously shown in the works of [69,70], fatty acids tended to be partitioned into the lipid bilayer. Thus, we also incorporated LA inside the model SC membranes in the beginning of simulations to decrease the simulation time required for proper equilibration of bilayers. The simulations were carried out for four types of systems in total: without any CPEs, with linoleic acid, with ethanol (50% molar solution), and with both types of investigated CPEs, i.e., linoleic acid and ethanol (see Table 1). 

**Figure 2:**
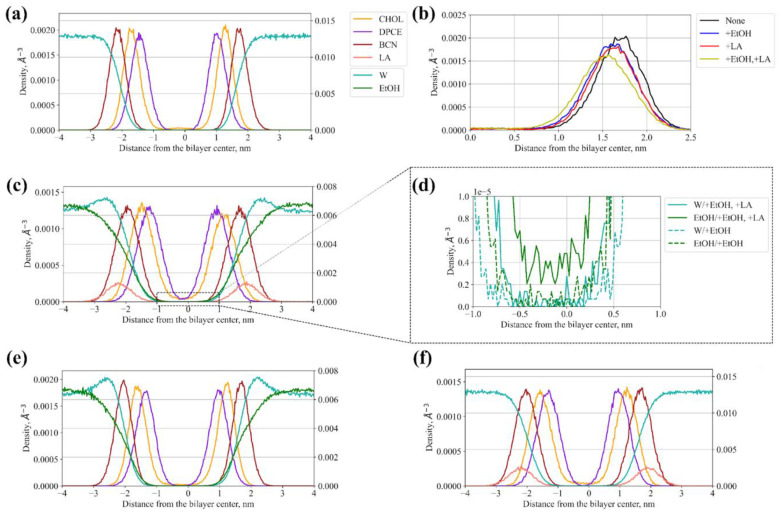
Density profiles of individual components of SC membranes, CPEs, and water for systems with and without CPEs: (**a**) SC bilayer without CPEs (the color code is the same for panels c, e, and f); (**b**) Density profiles for cholesterol without CPEs, with individual CPEs, and with their combination; (**c**) SC bilayer with both ethanol and linoleic acid; (**d**) Enlarged density of ethanol and water inside the hydrophobic region of SC membranes simulated with both ethanol and linoleic acid (solid line) and only with ethanol (dashed line); (**e**) SC bilayer with both ethanol; (**f**) SC bilayer with linoleic acid.

The representative density distributions of each penetration enhancer across the bilayer normal in different systems are shown in Figure 2.

The corresponding interleaflet distances measured between average positions of the hydrophilic P1, Qa, and SP1 CG beads (see Figure 1) of DPCE, behenic acid, and cholesterol, respectively, in two SC monolayers are provided in Table 2. None of the CPEs led to disintegration or large reshaping of SC at the simulated timescale, with all membrane components showing characteristic two-peak density profiles across the bilayer normal (Figure 2a,c,e,f). However, as one can see, the addition of both the LA and EtOH decreased the thickness of the membrane. EtOH decreases the interleaflet distance for the DPCE and CHOL by about ~2 Å and by ~1 Å for the behenic acid while LA decreases the distances by ~1 Å. Acting together, LA and EtOH induced the largest change in the SC thickness. The same trend is also clear from the density profiles plotted for all four investigated systems (Figure 2b).

Interestingly, we observed penetration of EtOH inside the hydrophobic core of SC in the simulation with both CPEs but not with EtOH (Figure 2d), indicating that LA facilitates partitioning of this compound inside SC presumably due to fluidization of the membrane.

**Figure 3:**
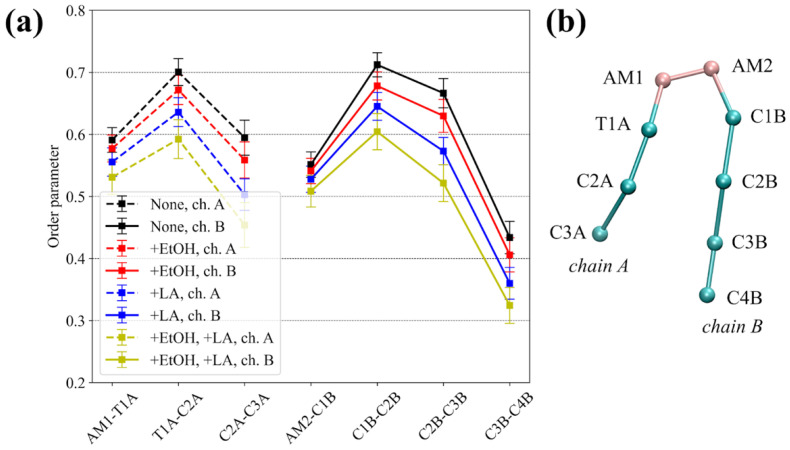
(**a**) Order parameter of the hydrocarbon tails of sphingosine (chain A) and the fatty acid (chain B) of the DPCE ceramide. ‘None’ corresponds to the system without enhancers, ‘+EtOH’, ‘+LA’, and ‘+EtOH, +LA’ to systems with either one or both enhancers added; (**b**) Scheme illustrating the coarse-grained representation of DPCE.

The order parameters of the hydrocarbon tails of sphingosine (chain A) and the fatty acid (chain B) of the DPCE ceramide were also calculated for all four MD systems (Figure 3). The addition of either EtOH or LA decreased the order parameters for both chains, which occurs, to a larger extent, in the case of LA, presumably due to the unsaturated nature of its acyl chain. Again, when acting together, CPEs decreased the order parameters to an even larger degree (Figure 3, yellow lines). 

**Figure 4:**
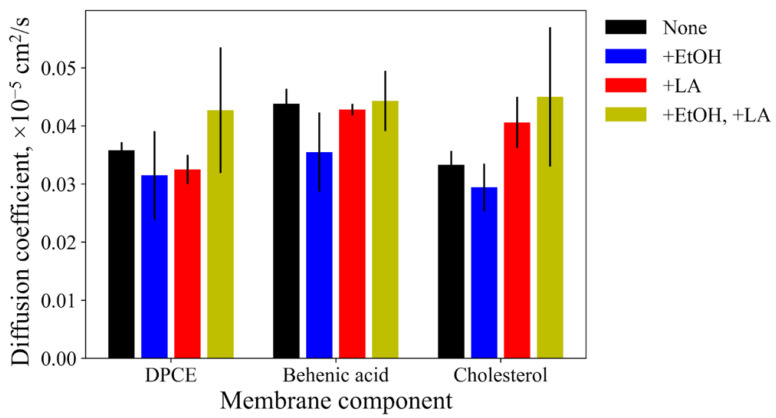
Diffusion coefficients of the individual membrane components: DPCE, behenic acid, and cholesterol. ‘None’ corresponds to the system without enhancers, ‘+EtOH’, ‘+LA’, and ‘+EtOH, +LA’ to systems with either one of both enhancers added.

The lateral diffusion coefficients for the membrane components are shown in Figure 4. EtOH slightly decreased diffusion coefficients for all of them, i.e., ceramide, cholesterol, and fatty acid. Contrarily, LA increases the diffusion coefficients for cholesterol and DPCE without any noticeable effect on linoleic acid. The addition of both enhancers to the system led to even larger diffusion coefficients for DPCE and cholesterol while the diffusion of behenic acid remained at the same level. At the same time, the dispersion of diffusion coefficients also increases in this case, as indicated by larger values of the standard deviation, implying significant disordering of the SC lipids.

Taking all these observations together, it appears that addition of 50% EtOH or LA affect the membrane thickness and order parameters of the hydrocarbon chains of DCPE. LA additionally affects the diffusion coefficients of uncharged SC bilayer components. The combination of two CPEs makes these alterations of SC properties more pronounced. 

### 3.2. PMF Calculations

**Figure 5:**
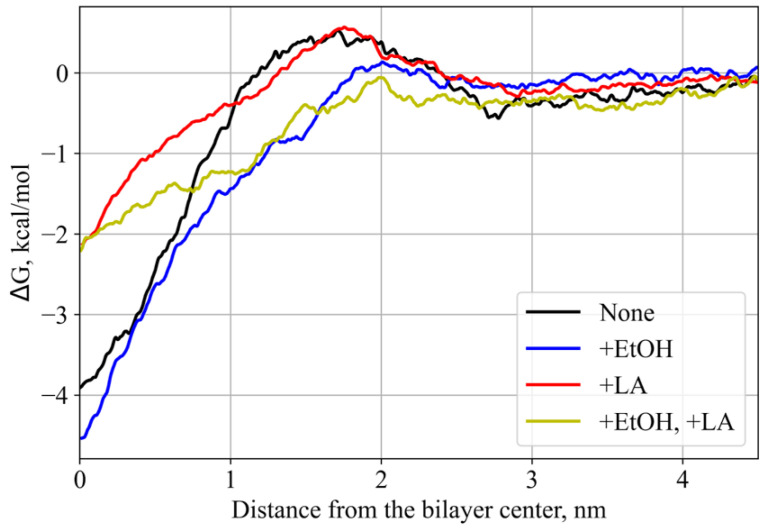
PMFs of lidocaine translocation across the SC membrane along the normal direction. ‘None’ corresponds to the system without enhancers, ‘+EtOH’, ‘+LA’, and ‘+EtOH, +LA’ to systems with either one or both enhancers added.

For energetical characterization of the test compound (lidocaine) permeation, we have estimated four potentials of mean force (PMF) for the process of its translocation across SC. PMF profiles (Figure 5) demonstrated that addition of EtOH decreased the energetic barrier for the lidocaine entrance inside the hydrophobic region of SC by ~0.6 kcal/mol. At the same time, the addition of LA decreases the depth of the global free energy minimum for lidocaine inside the SC membrane by ~2 kcal/mol, suggesting that it should facilitate the overall penetration of lidocaine across SC. Addition of two CPEs resulted in both features: decrease of the barrier for the SC entry and decrease of the global free energy minimum, implying a mechanism for the synergistic effect of this CPE combination.

## 4. Discussion

It has been proposed that the mechanisms of action of ethanol as CPE involve increasing the permanent concentration and affecting the lipid domains in the SC membranes [30,37,71,72]. Fatty acids are often used with cosolvents as they act synergistically to enhance the penetration of a drug [73,74]. It was shown that the higher the degree of unsaturation led to a more pronounced enhancing effect; moreover, the cis-conformation of unsaturated fatty acids led to a higher level of disruption of SC lipids compared to the trans-conformation [73]. It was also demonstrated that larger distances between the carboxylic group of fatty acid and its double bond(s) led to a higher drug flux [75].

Here, we investigated the effects of both types of abovementioned CPEs on properties of the model SC membrane containing DPCE, behenic acid, and cholesterol, and permeation of lidocaine across it. The equilibrium simulation conducted in the presence of 50% ethanol demonstrated that the membrane became thinner (Table 2, Figure 2b), the order parameters for DPCE slightly decreased (Figure 3), and the diffusion coefficients for all components also decreased (Figure 4). We did not observe the permeation of EtOH directly to the center of the SC membrane at the times up to 1 microsecond (Figure 2d). However, the simulation indicated that the ethanol molecules penetrated to the lipid headgroups and could even reach the area of the lipid tails (Figure 2e). Formation of favorable interactions between EtOH and headgroups of SC lipids was previously discussed in [30,37] and it apparently leads to slightly decreased diffusion coefficients of SC components and, at the same time, decreased free energy barrier for the entrance of lidocaine inside the hydrophobic region of SC since unionized lidocaine is much better soluble in EtOH compared to water. The latter effect apparently explains the penetration’s enhancing effect of ethanol at the explored concentration of 50%. In our simulations, the addition of EtOH did not result in water flow across the membrane (Figure 2d), or any other crucial disruptions of the membrane integrity as it was shown previously in [30,37]. It should be pointed out, however, that in the latter studies, the membrane disintegration was observed at much higher concentrations of ethanol up to 100%. It is worth mentioning that at higher concentrations, other mechanisms of penetration enhancement may become involved, e.g., formation of transmembrane pores [76] or denaturation of membrane proteins [77].

The addition of the unsaturated linoleic acid to the SC membrane in our simulations led to significant decreased order parameters (Figure 3 and Figure 4) and thickness of the membrane (Table 2). This observation is in good agreement with experimental and computational data [69,73]: two unsaturated carbon bonds led to more disturbance in the lipid bilayer, its fluidization and shortening of the hydrophobic region of SC. The diffusion coefficients increased only in the case of cholesterol, and slightly decreased in the case of DPCE but did not affect the behenic acid. This effect can be explained by the additional interactions of the charged fatty acid with DPCE and CHOL, and repulsion with the negatively- charged SC component, i.e., behenic acid. The free energy profile for lidocaine translocation across SC in the presence of linoleic acid revealed that the free energy barrier at the water-lipid interface remained the same but, instead, the depth of the global PMF minimum corresponding to the center of hydrophobic SC core decreased due to the less ordered and also less density of packed hydrocarbon chains [69,70]. In this situation, lidocaine can easily escape from the free energy well and eventually pass across SC.

Finally, the addition of both enhancers to the system resulted in more pronounced fluidization of the SC membrane as indicated by the DPCE order parameters. The thickness of the bilayer decreased by 0.25 nm and the dispersion of diffusion coefficients also increased, additionally implying a significant level of SC disordering. In this case, EtOH could also penetrate inside the hydrophobic core of SC (see Figure 2d). PMF profile for lidocaine translocation featured both effects that were observed earlier for individual CPEs: both the depth of the minimum in the SC center and the maximum at the headgroup interface decreased, resulting in increased permeability.

It is also worthwhile to mention that while the current analysis is limited solely to a combination of two prototypical CPEs, ethanol and linoleic acid, these results may be transferred to a broader group of related chemicals, including analogs of the present CPEs: oleic acid, lauric acid, and propylene glycol, which are also commonly used in combinations [14].

## 5. Conclusions

In the present study, we have mechanistically and energetically characterized the permeation mechanism of lidocaine across stratum corneum in the presence of two enhancers, linoleic acid and ethanol, as well as their combination by means of coarse-grained MD simulations. We demonstrated that both CPEs decreased the DPCE ordering in SC and its thickness, making SC more fluidic. However, these effects were more pronounced for linoleic acid. Both enhancers also affected the energetics of lidocaine penetration across the membrane: the addition of ethanol resulted in the decreased free energy barrier for the entrance of the compound inside the hydrophobic core of SC while the addition of linoleic acid decreased the depth of free energy minimum inside the lipid bilayer, facilitating the lidocaine passage across SC. Combination of both enhancers resulted in the synergistic effects on membrane fluidity reflected in larger decrease of order parameters, membrane thickness, and increase of diffusion coefficients. Moreover, the alteration of the free energy profile for the lidocaine translocation across SC resembled in this case are both characteristic features observed for individual enhancers. These results provide a mechanistic picture of synergistic action of penetration enhancers at molecular level. Future research should focus on increasing the range of tested concentrations, and the complexity of model SC membranes and CPEs’ formulations. We also believe that the developed models and methodology can be used to design or test various combinations of permeation enhancers in different drug or cosmetic formulations.

## Figures and Tables

**Table 1:** The list of simulated systems. CHOL—cholesterol; DCPE—N-Stearoylsphingosine; BCN—behenic acid; LA—linoleic acid; EtOH—ethanol; LID—lidocaine.

#	System Composition: Name/Molecules	Simulation Type	Total Simulation Time, μs
1	SC membrane (208 CHOL + 209 DPCE + 208 BCN) 1:1:1 + 21740 PW + 397 Na^+^ + 81 Cl^−^	Equilibrium	1
2	SC membrane (208 CHOL + 209 DPCE + 208 BCN + 57 LA) 18:18:18:5 + 21729 PW + 419 Na^+^ + 59 Cl^−^	Equilibrium	1
3	SC membrane (208 CHOL + 209 DPCE + 208 BCN) 1:1:1 + 10870 PW + 10870 EtOH + 397 Na^+^ + 81 Cl^−^	Equilibrium	1
4	SC membrane (208 CHOL + 209 DPCE + 208 BCN + 57 LA) 18:18:18:5 + 10870 PW + 10870 EtOH + 419Na^+^ + 59 Cl^−^	Equilibrium	1
5	SC membrane (208 CHOL + 209 DPCE + 208 BCN) 1:1:1 + 1 LID + 21740 PW + 397 Na^+^ + 81 Cl^−^	PMF	0.46
6	SC membrane (208 CHOL + 209 DPCE + 208 FFA + 57 LA) 18:18:18:5 + 1 LID + 21729 PW + 419 Na^+^ + 59 Cl^−^	PMF	0.47
7	SC membrane (208 CHOL + 209 DPCE + 208 FFA) 1:1:1 + 1 LID + 10870 PW + 10870 EtOH + 397 Na^+^ + 81 Cl^−^	PMF	0.46
8	SC membrane (208 CHOL + 209 DPCE + 208 BCN + 57 LA) 18:18:18:5 + 1 LID + 10870 PW + 10870 EtOH + 419 Na^+^ + 59 Cl^−^	PMF	0.47

**Table 2:** Interleaflet distance measured between average positions of P1, SP1, and SP1 CG beads of DPCE, behenic acid, and cholesterol, respectively, in two SC monolayers.

System	Interleaflet Distance, Å		
	DPCE	Behenic Acid	Cholesterol
None	24.78	38.55	30.54
+EtOH	22.53	37.55	28.54
+LA	23.28	37.80	29.54
+EtOH, +LA	22.03	36.30	27.28

## Data Availability

Not applicable.
